# Pluronic F127 “nanoarmor” for stabilization of Cowpea mosaic virus immunotherapy

**DOI:** 10.1002/btm2.10574

**Published:** 2023-07-14

**Authors:** Matthew D. Shin, Eunkyeong Jung, Miguel A. Moreno‐Gonzalez, Oscar A. Ortega‐Rivera, Nicole F. Steinmetz

**Affiliations:** ^1^ Department of NanoEngineering University of California La Jolla California USA; ^2^ Center for Nano‐ImmunoEngineering University of California La Jolla California USA; ^3^ Department of Bioengineering University of California La Jolla California USA; ^4^ Department of Radiology University of California La Jolla California USA; ^5^ Moores Cancer Center University of California La Jolla California USA; ^6^ Institute for Materials Discovery and Design, Department of NanoEngineering University of California La Jolla California USA

**Keywords:** compounds/materials, drug delivery, immunotherapies

## Abstract

Our lab demonstrated that intratumoral Cowpea mosaic virus (CPMV) is a potent antitumor immunotherapy when used as in situ vaccine. As we pave the way for human clinical translation, formulation chemistry needs to be optimized for long‐term storage of the drug candidate. In this work, CPMV was nanoengineered with Pluronic F127 to realize liquid and gel formulations which mitigate structural changes and RNA release during long‐term storage. We evaluated the CPMV‐F127 formulations for their stability and biological activity through a combination of in vitro assays and efficacy in vivo using a B16F10 murine melanoma model. Results demonstrate that both F127 liquid and gel formulations preserve CPMV structure and function following extended periods of thermal incubation at 4°C, 25°C, and 37°C. Heat‐incubated CPMV without formulation resulted in structural changes and inferior in vivo efficacy. In stark contrast, in vivo efficacy was preserved when CPMV was formulated and protected with the F127 “nanoarmor.”

## 1 INTRODUCTION

Plant viral nanoparticles (VNPs) are naturally evolved nanocarriers of genetic payloads and VNPs have been developed as vaccines and immunotherapy applications capitalizing on their immunomodulatory nature.^1^ The viral RNA genome and proteinaceous capsids function as pathogen‐associated molecular patterns (PAMPs) to trigger and activate innate immune cells. Building on these properties, our laboratory has developed Cowpea mosaic virus (CPMV) as an in situ vaccine. We discovered that when administered intratumorally, the plant virus stimulates both local and systemic antitumor immunity.^2^, ^3^ Potent and durable efficacy was demonstrated multiple tumor mouse models, and more importantly in companion canine cancer patients.^4^, ^5^ Mechanistic work demonstrates that the plant virus interfaces with the immune system through multiple axes and with multivalency—therefore, the multipronged immunomodulation results in a cascade of events boosted by avidity effects—thus achieving unprecedented potency.^6^, ^7^ The plant virus in situ vaccine induces durable antitumor immunity to prevent cancer recurrence with demonstrated abscopal effect as shown by reported systemic efficacy on untreated, distant metastatic tumors.^3^

**FIGURE 1:**
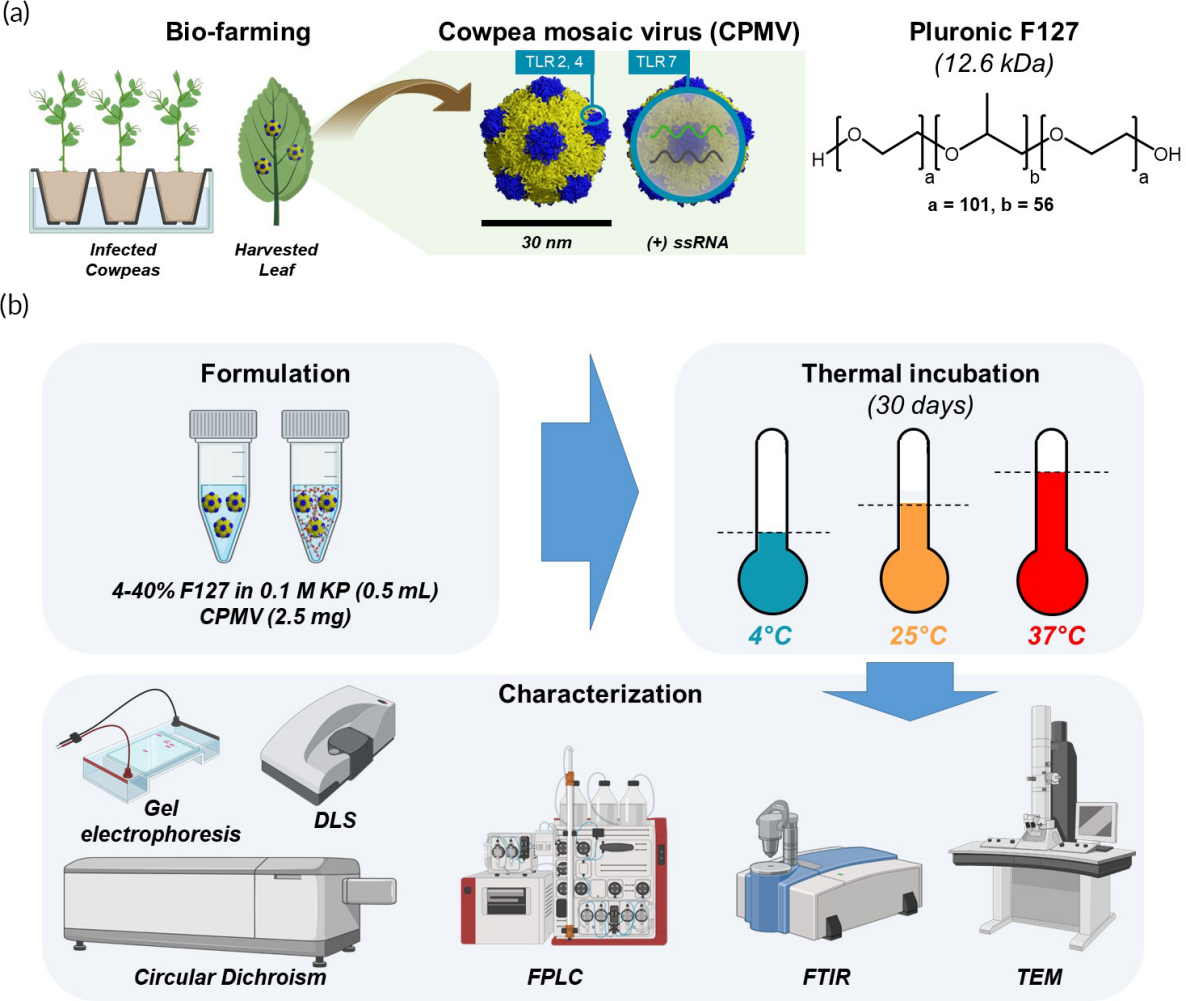
Preparation, formulation, and characterization of CPMV‐F127 liquid and gel formulation. (a) Schematic illustration of the propagation and harvesting of CPMV from cowpeas; the structure of CPMV and its immunogenic coat protein and RNA; the chemical structure of Pluronic F127. (b) CPMV formulation compositions, samples subject to thermal challenge, and their subsequent characterization. CPMV, cowpea mosaic virus; DLS, dynamic light scattering; FPLC, fast protein liquid chromatography; FTIR, Fourier‐transform infrared spectroscopy; F127, Pluronic F127; KP, potassium‐phosphate buffer; TEM, transmission electron microscopy; TLR, toll‐like receptor; (+) ssRNA, positive‐strand ribonucleic acid. Graphics generated using Biorender.com.

In addition to pharmacology and toxicology evaluation, formulation chemistry must be optimized to pave the way for clinical translation. In this work, we focused on the formulation of CPMV with Pluronic F127 as a means to stabilize CPMV structure during long‐term storage to preserve biological activity. CPMV is a ~30 nm icosahedral nanoparticle with pseudo *T* = 3 symmetry whose capsid is composed of 60 asymmetrical units, each composed of small (24 kDa) and large (42 kDa) coat protein (Figure 1a). The capsid is self‐assembled around a bipartite (+)‐sense ssRNA. Data indicate that both the viral RNA and capsid are required to achieve optimal potency as in situ vaccine.^7^, ^8^, ^9^ The protein components of CPMV signal through toll‐like receptor (TLR) 2 and 4 and its ssRNA acts as a TLR7 agonist.^7^ While various reports note the good thermostability of CPMV,^10^, ^11^ no detailed stability studies have been reported. Therefore, we studied the stability of CPMV in storage buffer at three temperatures, 4°C, 25°C, and 37°C. 37°C was used to accelerate protein degradation (Figure 1b). Additionally, we explored the thermostability of CPMV when formulated with Pluronic F127 (Figure 1a), a thermoresponsive pharmaceutical excipient, which we previously utilized to enhance bioconjugation between CPMV and hydrophobic ligands.^12^ Pluronic F127 coatings have been applied as stabilizers for biologics used as vaccines or tissue engineering^13^, ^14^, ^15^, ^16^, ^17^, ^18^, ^19^; the application in the context of the CPMV in situ vaccine is novel. Native and F127 “nanoarmored” CPMV were stored at 4°C, 25°C, and 37°C. Following a 30 day temperature incubation, liquid and gel CPMV‐F127 formulations were characterized for their structural integrity and biological activity in vitro using RAW‐Blue™ reporter cells; antitumor efficacy was then tested using a dermal murine melanoma model (using B16F10 cells and C57BL/6 mice).

## 2 MATERIALS AND METHODS

### 2.1 Reagents and cells

Pluronic F127 (F127) was obtained from Sigma–Aldrich (St Louis, MO, USA). Dibasic monohydrogen phosphate and monobasic dihydrogen phosphate and were purchased from Thermo Fisher Scientific (Waltham, MA, USA). Cowpea mosaic virus (CPMV) was propagated in plants and harvested using previously reported biopharming techniques.^20^ Purified CPMV material was stored in 0.1 M potassium phosphate (KP) buffer (pH 7.0). Pure CPMV was quantified via UV/vis spectroscopy (CPMV *ε*_260nm_ = 8.1 mg^−1^ mL cm^−1^). Virus integrity was confirmed by FPLC, DLS, and TEM (see below). RAW‐Blue™ cells (Invivogen, CA, USA) were cultured and maintained in selection media bearing Normocin (Invivogen, CA, USA) and Zeocin (Invivogen, CA, USA) according to manufacturer instructions. B16F10 mouse skin melanoma cells were purchased from ATCC (CRL‐6475), cultured, and maintained in Dulbecco's modified Eagle's medium (DMEM) supplemented with 1% (v/v) penicillin/streptomycin and 10% (v/v) fetal bovine serum (FBS) at 37°C in a 5% CO_2_ chamber. Both DMEM and PBS were purchased from Corning Life Sciences. Penicillin/streptomycin (P/S) was purchased from ThermoFisher Scientific. Fetal bovine serum (FBS) was purchased from Atlanta Biologicals.

### 2.2 “Nanoarmoring” CPMV via formulation with Pluronic F127

CPMV was mixed with 4% (CPMV‐4P), 16% (CPMV‐16P), 23% (CPMV‐23P), 30% (CPMV‐30P), or 40% (CPMV‐40P) (w/w) F127 on ice, resulting in a sample containing a total volume of 0.5 mL of 0.1 M KP buffer and a total protein concentration of 5 mg/mL. To ensure homogeneous distribution of material, samples were incubated on a rotator overnight at 4°C prior to employment in thermal incubation assay.

### 2.3 Thermal incubation assay

CPMV “nanoarmor” formulations were incubated at 4°C, 25°C, and 37°C for 14 or 30 days in 0.5 mL of 0.1 M KP buffer. Samples were incubated using a ThermoMixer F2.0 apparatus (Eppendorf, MA, USA). At the end of the incubation period, samples were transferred to a water‐ice bath for at least 5 min prior to preparing samples for downstream characterization by agarose, FPLC, DLS, CD, SEC, TEM/SEM, and FT‐IR (see below).

### 2.4 Agarose gel electrophoresis

1.2% (w/v) agarose gels were run for 30 min at 120 V and 400 mA in 1× tris‐acetate‐EDTA (TAE) running buffer in the presence of GelRed Nucleic Acid Gel Stain (GoldBio) diluted 1:10,000 (v/v). 10 μg of CPMV formulations were prepared in 0.1 M KP along with 6× Gel Loading Purple dye (Biolabs) and analyzed in sample wells. Gels were imaged before and after staining with 0.25% (w/v) Coomassie Brilliant Blue G‐250 from Sigma Aldrich (St Louis, MO, USA) using the ProteinSimple FluorChem R imaging system (MN, USA) under UV light and white light, respectively.

### 2.5 Fast protein liquid chromatography (FPLC)

FPLC apparatus ÄKTA pure 25 M1 (Cytiva, MA, USA) equipped with a 10 × 300 Superose 6 size exclusion column (Cytiva, MA, USA) was used to characterize structural integrity and purity of CPMV samples. Runs were performed at a flow rate of 0.5 mL/min under isocratic elution for 50 mL. Samples were diluted to 0.3 mg/mL using 0.1 M KP buffer, injected into the column, and monitored using absorbance measurements at 260 and 280 nm to interrogate nucleic acid and protein concentrations, respectively. The 260:280 absorbance ratio at the elution peak was calculated for each sample to monitor CPMV structural integrity.

### 2.6 Dynamic light scattering (DLS)

Samples were diluted to 1 mg/mL in 0.1 M KP buffer and then subsequently measured using a Zetasizer Nano ZSP/Zen 5600 instrument (Malvern Panalytical, Malvern, UK) to determine hydrodynamic diameter. *Z*‐average was calculated as the weighted mean of the intensity distribution.

### 2.7 Circular dichroism (CD)

CD spectra were measured using an Aviv model 215 CD spectrometer (AVIV Associates, Lakewood, NJ) in a 0.1 M KP buffer in a quartz cuvette with 2 mm pathlength. CD samples were prepared by diluting thermally incubated samples into additional KP buffer to a final protein concentration of 0.33 mg/mL and a total volume of 0.4 mL. Spectra were recorded from 185 to 260 nm with 0.5 nm intervals and an averaging time of 1 s at 25°C.

### 2.8 TEM

CPMV samples were diluted to 0.2 mg/mL in Milli‐Q water, loaded onto FCF400‐CU 400‐mesh copper grids (Electron Microscopy Sciences, Hatfield, PA, USA), and imaged using a FEI Tecnai Spirit G2 BioTWIN TEM (Hillsboro, OR, USA) at 80 kV. 10 μL samples were loaded for 2 min, then washed with 10 μL Milli‐Q water twice for 30 s, and finally stained with 10 μL uranyl acetate (2% w/v) for 1 min.

### 2.9 FTIR spectroscopy

Fourier Transform Infrared (FT‐IR) measurements were performed using a PerkinElmer Spectrum Two FT‐IR Spectrometer with Universal ATR Sampling Accessory (Perkin Elmer, Waltham, MA, USA). First, the blank formulation buffer was loaded, and a background spectrum was collected. Next, 20 μL per formulation, in matching buffer, were loaded and corresponding sample spectra were collected; CPMV formulations had a concentration of 10 mg/mL. Spectra were recorded over the range of 2000–1200 cm^−1^ with 64 scans. Data was recorded in transmittance, replotted as absorbance, and second derivative spectra of absorbance were generated using PerkinElmer Spectrum software algorithms. Exported data was processed and plotted using Spectragryph; this software was used to identify the 2nd derivative peaks from 1800 to 1500 cm^−1^ (Amide I region) of the spectra, which were used to interpret protein secondary structure.

### 2.10 RAW‐Blue™ cell activation assay

RAW‐Blue™ cells were maintained at 37°C in a 5% CO_2_ atmosphere and grown to 70% confluency in DMEM supplemented with 10% (v/v) fetal bovine serum, 1% (v/v) penicillin/streptomycin, and 100 μg mL^−1^ of Normocin and Zerocin per manufacturer instructions.

Cells were then washed twice with PBS and collected with a cell scraper. Cells were pelleted at 500 g for 5 min and resuspended in 1 mL of test medium (DMEM with l‐glutamine supplemented with 10% (v/v) heat inactivated FBS and 1% (v/v) penicillin–streptomycin). Cells were seeded on a 96‐well tissue plate (100,000 cells in 180 μL media per well), then 20 μL of CPMV sample was added (1 μg CPMV). After 24 h of incubation, 20 μL of the supernatant from each well were mixed with 180 μL of Quanti‐Blue solution (Invivogen, CA, USA) and then incubated for 2 h at 37°C. Absorbance readout (OD655 nm) was measured using a Tecan Infinite M200 microplate reader (Tecan Group Ltd., Switzerland).

### 2.11 B16F10 melanoma model and in situ vaccination

All mouse studies performed were approved by and conducted in accordance with the Institutional Animal Care and Use Committee of the University of California, San Diego (protocol number S18021). First, B16F10 cells were cultured at 37°C and 5% CO_2_ in Dulbecco's Modified Eagle Medium (DMEM) with l‐glutamine, supplemented with 1% (v/v) penicillin/streptomycin and 10% (v/v) fetal bovine serum (FBS). Upon reaching 80% confluence, cells were subjected to trypsin digest and harvested via centrifugation at 150 × *g* for 5 min. Next, cells were resuspended in PBS and 2.5 × 10^5^ B16F10 cells in 30 μL were orthotopically implanted via intradermal injection on the right flank of 6–8 weeks old female C57BL/6 mice purchased from The Jackson Laboratory (strain number 000664). Tumors were treated using 100 μg of CPMV, CPMV‐4P, and CPMV‐40P, stored at 4°C, 25°C, and 37°C for 30 days, administered via intratumoral or in situ injection in 20 μL of PBS. Weekly treatment was given starting ~day 9 post‐tumor inoculation when tumors reached ~60 mm^3^ in volume. Tumors were measured by digital caliper and tumor volume (mm^3^) was calculated using the following equation: *V* = (*L* × *W* × *W*)/2, where *V* is tumor volume, *L* is tumor length, and *W* is tumor width. Animals were euthanized when the tumor volume exceeded 1000 mm^3^. Tumor‐free survivors were rechallenged with 2.5 × 10^5^ B16F10 cells per mouse by intradermal injection on the contralateral flank; animals were evaluated until day 22 when tumors of age‐matched mice treated with PBS reached volumes of ~1000 mm^3^.

Although melanoma afflicts women and men, and therefore translational research should consider testing in female and male mice. In this work, we used only female C57BL6 mice with dermal tumors from B16F10 cells for the following reasons: in prior research, we demonstrated that CPMV in situ vaccination is indeed effective in male and female mice with B16F10 tumors; beyond this, we demonstrated efficacy in multiple tumor types at various anatomical locations as well as in pets with cancer. Because efficacy of CPMV was already established and because tumor growth rates of B16F10 melanoma varies in male vs. female mice, we chose only female mice to keep the animal numbers used to a minimum. Future translational work with the F127 “nanoarmored” CPMV must consider both sexes.

### 2.12 Statistics

Statistical significance denoted as *p* > 0.05 as ns, *p* < 0.05 as *, *p* < 0.01 as **, *p* < 0.001 as ***, and *p* < 0.0001 as **** in figures. Data for the bar graph are calculated using unpaired Student's *t*‐test.

Data are expressed as the mean ± standard error of the means (SEM), as indicated. Data for survival study are calculated using Student's *t*‐test to compare two groups and Sidak's or Tukey's multiple comparison tests was used to compare three or more groups. Survival rates were analyzed using the log‐rank (Mantel–Cox). Statistical analysis performed using GraphPad Prism v7.0 (GraphPad Software).

## 3 RESULTS AND DISCUSSION

### 3.1 Stability profiles of thermally‐challenged CPMV formulations

**FIGURE 2:**
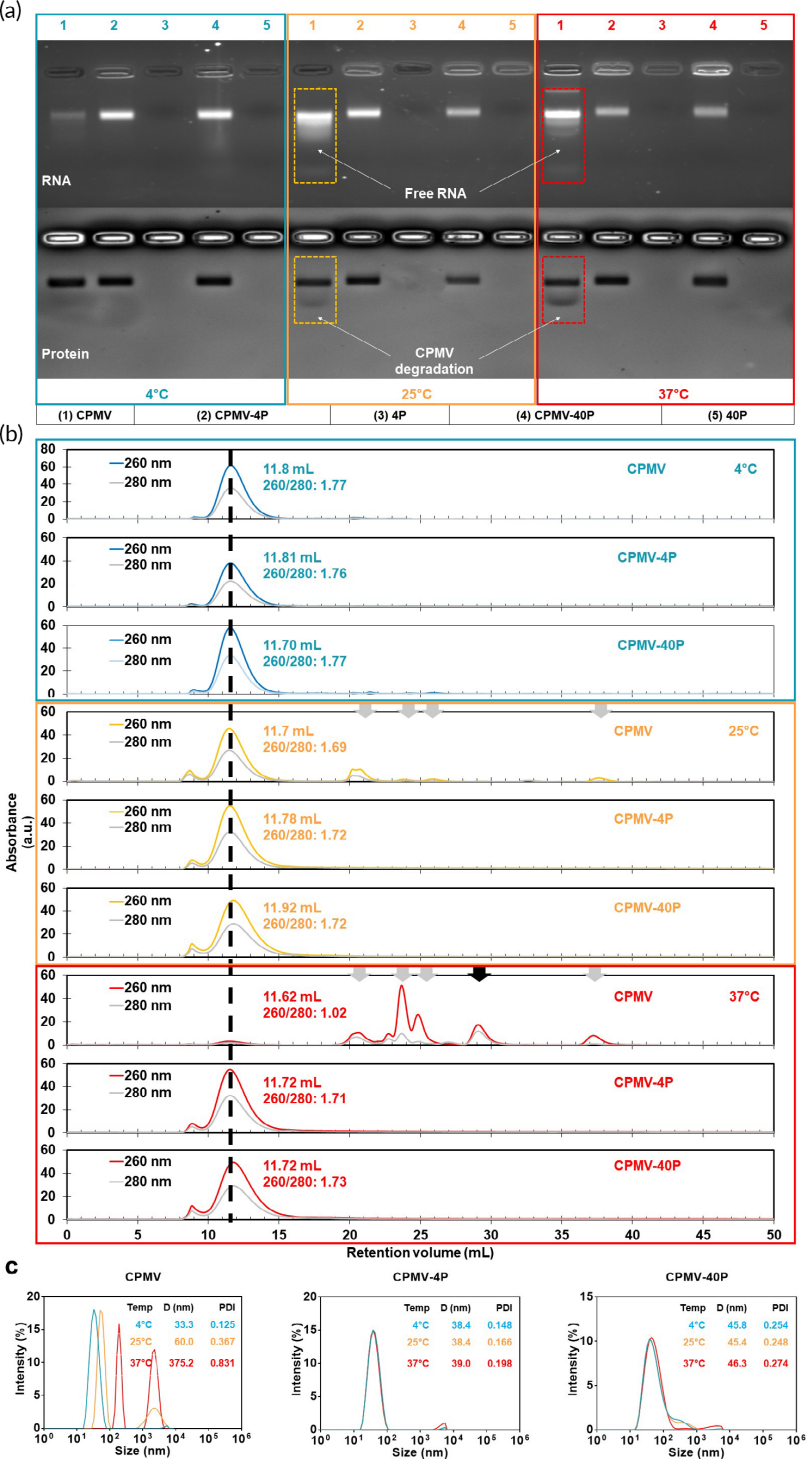
Characterization of CPMV and “nanoarmored” CPMV‐F127 post‐thermal challenge. (a) Agarose gel electrophoresis analysis of CPMV and CPMV‐F127 after 30‐day incubation at 4°C, 25°C, and 37°C; top image is GelRed stained for RNA and bottom image is Coomassie Blue stained for protein; lane 1—CPMV, lane 2—CPMV‐4P, lane 3—4P, lane 4—CPMV‐40P, lane 5—40P. (b) Size exclusion chromatography (SEC); A260:A280 ratio was determined at the peak elution volume indicated. (c) Dynamic light scattering (DLS) analysis (Z‐average and PDI data are mean values, *n* = 3).

The viral capsid's key function is to protect its genome against biological, chemical, and mechanical damage. Plant viruses are generally considered stable nanoparticles because they are evolved to withstand various environmental conditions. Nevertheless, we noted changes in structural integrity of CPMV when stored at elevated temperatures (see below). We therefore aimed to stabilize CPMV through formulation with F127. To assess the F127 effect on CPMV stability, we incubated native and F127 “nanoarmored” CPMV for 30 days at 4°C, 25°C, and 37°C. We assessed the effectiveness of 4% vs. 40% F127. When using 4% F127, the formulation, referred to as CPMV‐4P, remains a liquid at 25°C. While using 40% F127 leads to gelling resulting in a CPMV‐40P hydrogel at 25°C (Figure S1), which transitions to a liquid at 4°C. After 30 days of incubation, CPMV and CPMV‐F127 samples (CPMV‐4P and CPMV‐40P) were processed and analyzed via native agarose gel, size‐exclusion chromatography (SEC), and dynamic light scattering (DLS) which is summarized in Figure 2. Of note, due to its thermoresponsive nature, all 40P samples were first incubated on ice for 5 min to enable gel‐to‐sol transition, samples were then vortexed and transferred for analysis.

Native gel electrophoresis using agarose gels indicates that CPMV is stable at 4°C, but degradation is indicated when CPMV is incubated at 25°C (CPMV‐25C) and 37°C (CPMV‐37C). Imaging of the RNA and protein indicates loss of RNA and/or capsid degradation with higher mobility RNA and protein bands detected. The effects are more profound at 37°C vs. 25°C. In stark contrast, CPMV‐4P and CPMV‐40P remain intact with their electrophoretic mobility profile matching that of CPMV‐4C. This finding was further corroborated with SEC and DLS. Intact CPMV elutes from the Superose 6 Increase column at ~11.5 to 12 mL with a characteristic elution peak absorbance ratio at 260 nm and 280 nm (A260:A280) between ~1.7 and 1.8.

**FIGURE 3:**
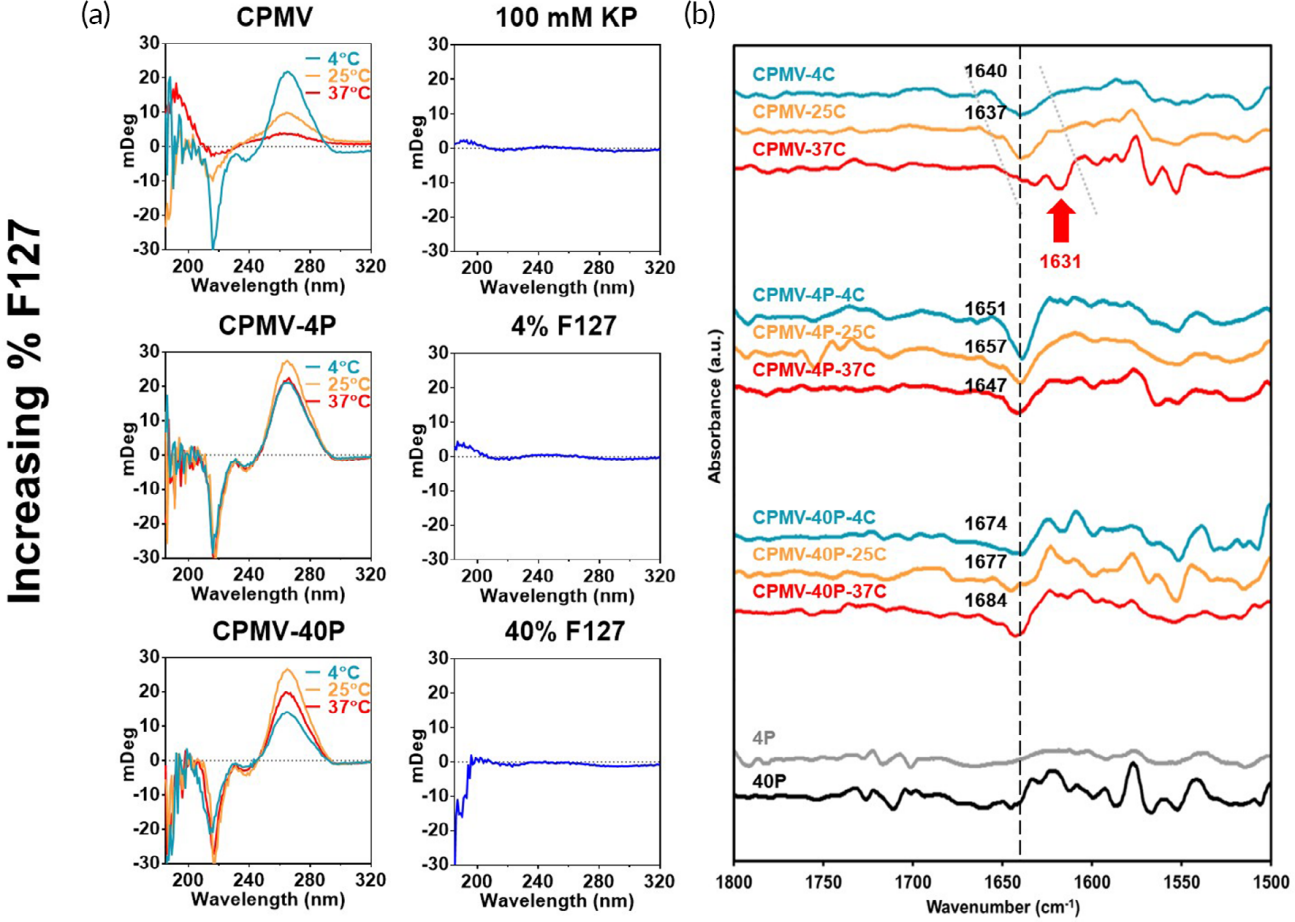
Secondary structure characterization of thermally challenged CPMV and CPMV‐F127 by Circular dichroism (CD). (a) CD spectra grouped by formulation type and protein‐free matrix controls. (b) FTIR 2nd derivative spectra were calculated from measured absorbance spectra and grouped by formulation type.

SEC profiling of native CPMV incubated at elevated temperatures were consistent with nanoparticle degradation: while only minor degradation products were noted for the CPMV‐25C sample, in the CPMV‐37C sample, the main peak was lost and only smaller degradation products were detectable which indicates that this sample is severely damaged (gray and black arrows, Figure 2b). In stark contrast, F127 “nanoarmored” formulations remained intact as indicated by SEC profiles generally matching that of stable CPMV‐4C. The main peak was slightly broader with a small leading peak at ~9 mL, which may be attributed to the F127 coating. Subtle indications of peak tailing were observed in CPMV‐F127 samples, which may be due to an increase in intermediate viscosity which is reported to produce such artifacts in Superose 6 Increase columns. DLS data were in agreement showing multimodal intensity profiles for the CPMV‐25C and CPMV‐37C particles, indicating aggregation and distinct populations with a *Z*‐average of ∼60.0 nm (PDI = 0.367) and ~375.2 (PDI = 0.831), respectively (Figure 3c). In contrast, as we formulate CPMV using the F127 “nanoarmor,” improvement in size stability and PDI for the corresponding temperature‐incubated samples is evidenced by narrower unimodal intensity profiles. The *Z*‐average of the CPMV‐4P particles was ~38.4 nm (PDI = 0.148) and CPMV‐40P particles was 45.8 nm (PDI = 0.254), both with a similar uniform, unimodal intensity profile compared to CPMV‐4C, albeit relatively larger than CPMV‐4C which measured a *Z*‐average of ~33.3 nm (PDI = 0.125). The observed size increase was directly proportional to F127 percentage increase, which may be attributed to the F127 “nanoarmoring” or coating effect on CPMV (Figure 3c). Together this data indicates instability of CPMV at elevated temperatures and suggest that F127 acts as a “nanoarmor” protecting CPMV structure.

### 3.2 Secondary structure characterization of thermally challenged native and F127 “nanoarmored” CPMV

CD is commonly used to analyze protein secondary structure; however, insights into tertiary structure may also be obtained. In the far‐UV (190–250 nm), CPMV‐4C displayed a negative peak ~218 nm which indicated significant β‐sheet structure. CPMV is composed of 60 asymmetric units, each bearing 3 β‐barrels—two in L and one in S protein for a total of 180 β‐barrels per virion.^21^ Quite strikingly, the 218 nm peak significantly diminished in a stepwise fashion as incubation temperature increased from CPMV‐25C to CPMV‐37C (Figure 3a). Additionally, the positive peak at ~280 nm in the near‐UV region (250–320 nm) significantly decreased in magnitude and broadened in width due to increasing heat incubation. The 280 nm peak can be attributed to tyrosine which plays a role in tertiary protein structure due to its *π*–*π* stacking interactions, hydrogen bonding, and electrostatic interactions with other amino acid residues, which can help to stabilize the protein fold and the virus particle.^22^ Notably, CPMV is host to 15 tyrosine residues.^23^ CD data are consistent with nanoparticle characterization (see Figure 2) and indicates that F127 protects CPMV against degradation as both the CPMV‐4P and CPMV‐40P sample groups have well‐preserved 218 nm and 280 nm peaks, overall matching the native CPMV peaks.

FTIR is employed in industry to probe the secondary structure of proteins, antibodies, and viruses.^24^ Based on FTIR analysis of human papillomavirus (HPV) Type 6 L1 protein virus‐like particle (VLP), we analyzed CPMV by taking the second derivative of the absorbance spectra. Given that the HPV VLP contains 60 β‐barrels and thus yielding a detectable amide I band C=O stretch, we had confidence CPMV would too yield signal due to its 180 β‐barrels.^25^ Indeed, using FTIR we detected a signal at 1640 cm^−1^ for CPMV‐4C, which is consistent with β‐sheet secondary signal (Figure 3b). The decrease in absorption from 1640 cm^−1^ to 1631 cm^−1^ observed for CPMV‐4C to CPMV‐37C, respectively, suggests a weakening of hydrogen bonding within CPMV capsids, where vibrational frequencies are decreasing due to aggregation and an increasing hydrophobic environment. By extension, this decrease in vibrational frequency also suggests a greater degree of intermolecular interaction versus intramolecular interaction, as was observed for HPV VLPs.^26^ The FTIR data are consistent with DLS data in that samples with greater intramolecular interaction, CPMV‐25C and CPMV‐37C, also displayed higher *Z*‐avg and PDI (Figure 2c). Of note, CPMV‐F127 formulations exhibited an increase in absorption from 1640 cm^−1^ to 1651 cm^−1^ to 1674 cm^−1^, which indicates F127 is facilitating stronger intramolecular character and increasing hydrogen bonding stoichiometry. One could speculate that the terminal PEO or poly(ethylene oxide) end groups of Pluronic F127 (Figure 1a) may be hydrogen bonding with solvent‐exposed amides or carboxylic acids along the protein backbone. In fact, it was demonstrated that F127‐coating of tobacco mosaic virus (TMV) was prevented if F127 PEO groups were masked with α‐cyclodextrin.^27^ However, it has also been demonstrated that F127 can coat synthetic hydrophobic surfaces through the association of its PPO or poly(propylene oxide) core group.^28^ A hydrophilic–hydrophilic, hydrogen bond‐driven mechanism of viral capsid stabilization by F127 is more likely; however, future work using molecular dynamics and/or cryo‐EM may provide a more conclusive analysis.

### 3.3 In vitro immunostimulation of CPMV‐F127

**FIGURE 4:**
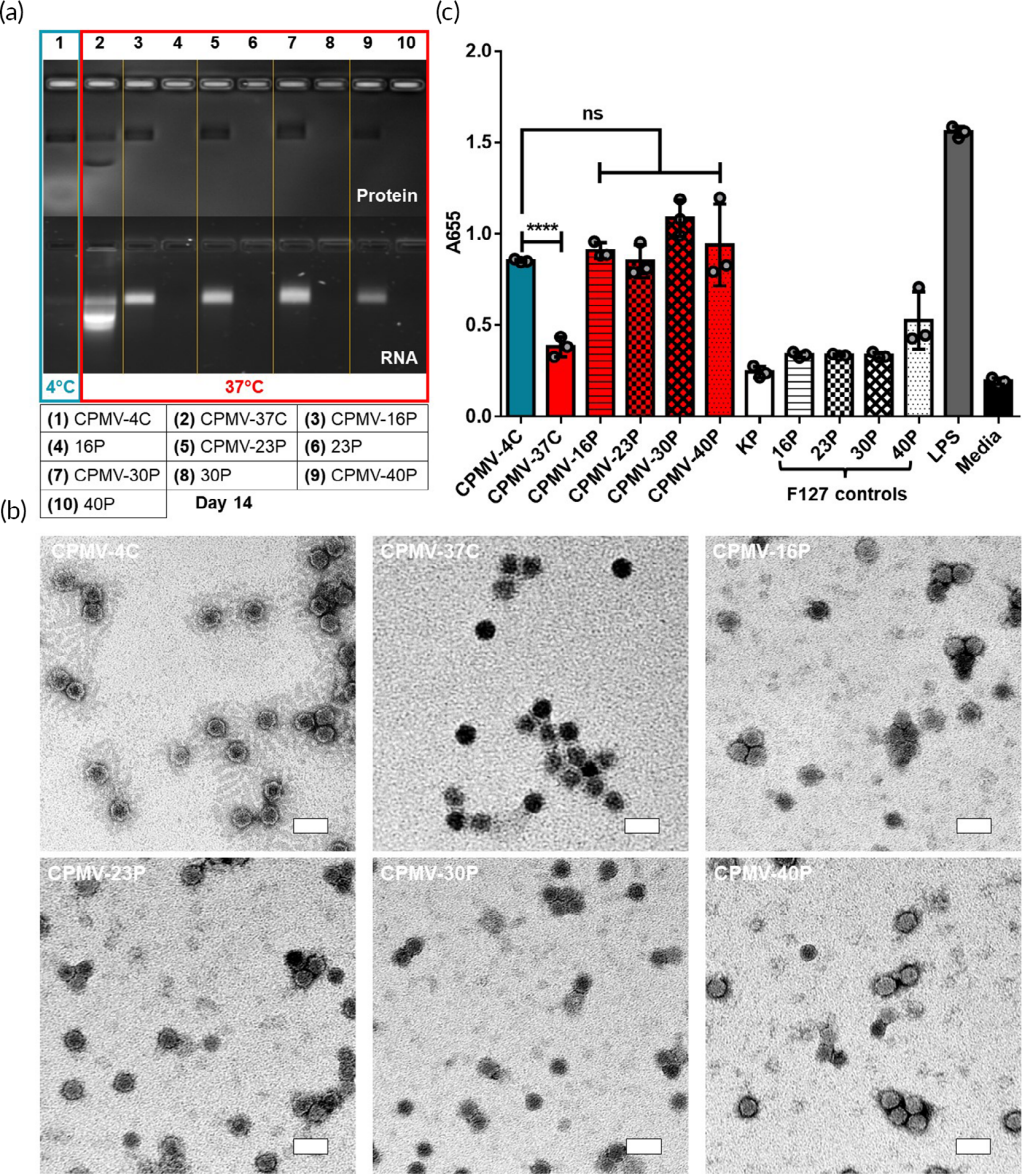
Morphological and functional evaluation of CPMV‐F127 hydrogels after thermal incubation. (a) Agarose gel electrophoresis analysis of the CPMV stored in various F127 hydrogel formulations (16%–40%) at 37°C for 14 days; lane 1 control—CPMV‐4C (no thermal incubation), lane 2—CPMV‐37C, lane 3—CPMV‐16P, lane 4—16P, lane 5—CPMV‐23P, lane 6—23P, lane 7—CPMV‐30P, lane 8—30P, lane 9—CPMV‐40P, lane 10—40P. (b) TEM images of CPMV particles stained with uranyl acetate. Scale bars represent 50 nm. (c) RAW‐Blue™ cell activation of CPMV particles. Results were compared using an unpaired *t*‐test (*p* > 0.05 as ns, *p* < 0.05 as *, *p* < 0.01 as **, *p* < 0.001 as ***, and *p* < 0.0001 as ****). Data are means ± SEM (*n* = 3).

CPMV is a TLR agonist and therefore we assayed its immunostimulatory properties using RAW‐Blue™ cells which were established to monitor the NF‐kB and AP‐1 responses upon pattern recognition receptor (PRR) stimulation; we have previously shown utility of these cells for assaying various viral nanoparticle formulations.^29^ Here we used CPMV formulated as hydrogels using F127 at concentration ranging from 16 to 40%. All formulations formed hydrogels at 37°C and samples were stored at 37°C for 2 weeks prior to analysis by native gel electrophoresis and TEM (Figure 4a,b). Of note, CPMV‐4C control was not incubated at 37°C. Both methods confirmed intactness of “nanoarmored” CPMV and showed degradation for the CPMV‐37 sample. Of note, CPMV‐37C displayed an intense positive stain on TEM, highlighting the accumulation of uranyl acetate (UAc) within the viral capsid. Increased UAc penetration may be due to morphological changes to the viral capsid and loss of the genetic material,^30^ that is, a more porous CPMV capsid and lack of RNA for degraded CPMV‐37C. Samples were then subjected to the RAW‐Blue™ reporter assay. Unformulated CPMV‐37C exhibited poor immunogenicity in RAW‐Blue™ reporter assay (Figure 4c). In contrast, CPMV‐F127 formulations showed no sign of degradation and retained their immunogenicity compared to CPMV‐4C control (Figure 4c). This data indicates that “nanoarmored” CPMV‐F127 formulations remain structurally sound and retain their biological activity.

### 3.4 In situ vaccine efficacy of CPMV formulations in a murine B16F10 tumor model

**FIGURE 5:**
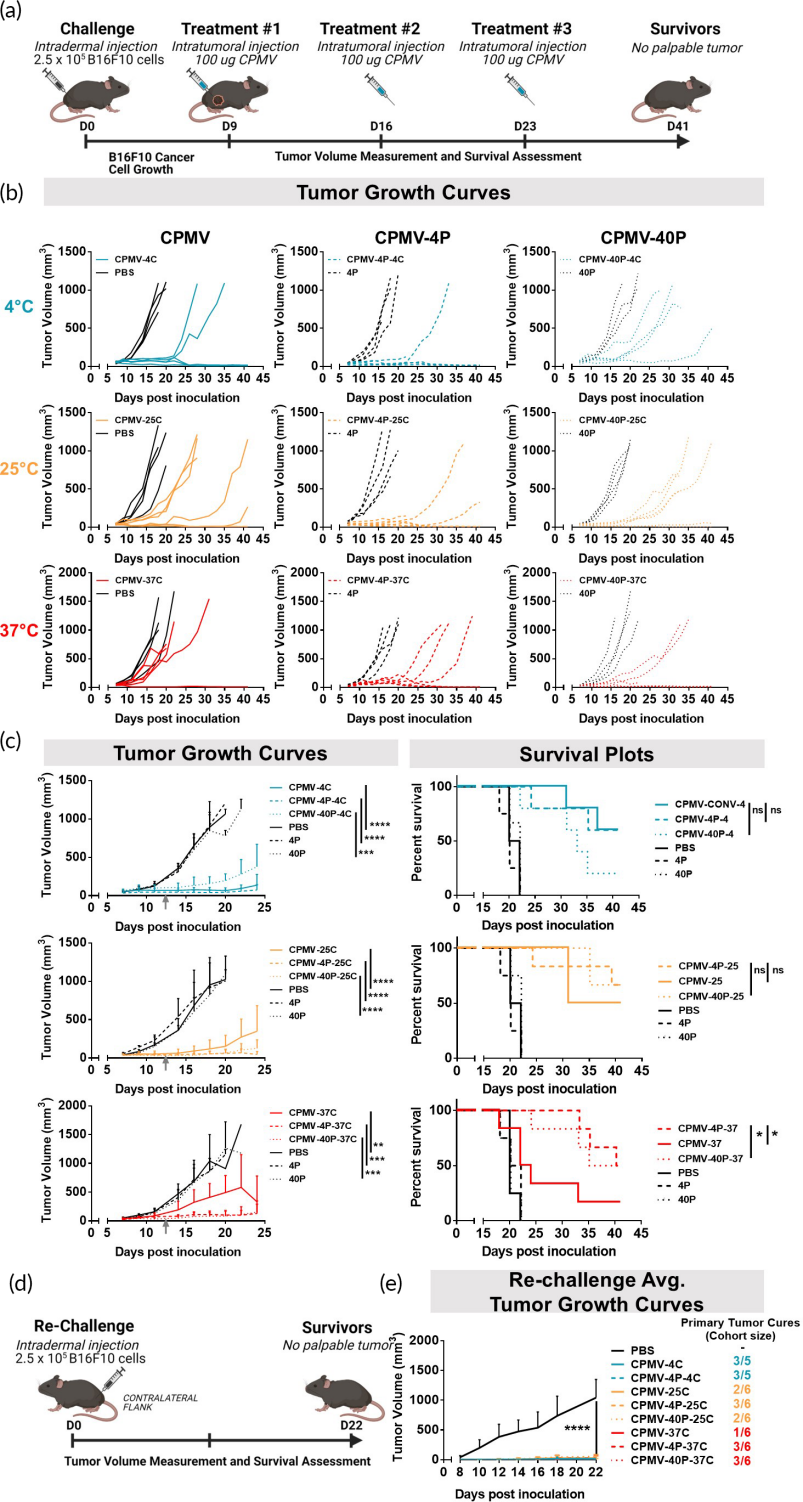
F127 formulation improves CPMV in situ vaccination efficacy for samples incubated at elevated temperatures. (a) Schematic representation of murine B16F10 melanoma model. C57BL/6 mice were intradermally injected with 250,000 B16F10 cells at day 0. CPMV, CPMV‐4P, and CPMV‐40P stored at 4°C, 25°C, and 37°C for 30 days were administered intratumorally on day 9, 16, and 23. (b) Individual tumor growth curves of mice bearing B16F10 melanoma. (c) Average tumor growth curves and survival plots. Results were compared using an unpaired *t*‐test (*p* < 0.05 as *, *p* < 0.01 as **, *p* < 0.001 as ***, and *p* < 0.0001 as ****). Data are means ± SEM (*n* = 3–6). Survival curves were compared using log‐rank (Mantel‐Cox) test (*p* > 0.05 as ns, *p* < 0.05 as *, *p* < 0.01 as **, *p* < 0.001 as ***, and *p* < 0.0001 as ****). (d) Schematic representation of rechallenge study using tumor‐free mice from (c), which were rechallenged with B16F10 33 days after first treatment in (b). (e) Tumor growth curves for rechallenged mice. Cohort sizes of mice cured of primary tumors ranged from *n* = 1–3 and PBS cohort size was *n* = 5. Data are means ± SEM (where *n* > 1). Results were compared using an unpaired *t*‐test between groups of at least *n* = 3 (*p* < 0.05 as *, *p* < 0.01 as **, *p* < 0.001 as ***, and *p* < 0.0001 as ****). Graphics generated using Biorender.com.

Next, we used a dermal mouse melanoma model (using B16F10 and C57BL/6 mice) to validate in vivo efficacy of F127‐formulated CPMV. C57BL/6 mice were inoculated intradermally on the flank with 2.5 × 10^5^ B16F10 cells per mouse. Once tumors reached ~60 mm^3^ (9 days post‐tumor challenge), mice were treated with three weekly intratumoral injections of CPMV, CPMV‐4P, or CPMV‐40P (100 μg VNP/20 μL PBS) stored at the 4°C, 25°C, and 37°C (samples were stored at the various temperatures for 30 days prior to this study) (Figure 5a). PBS, and free F127 (4P and 40P) treatments were used as controls.

By day 18, for samples incubated at 4°C, CPMV‐4C, treatment significantly inhibited tumor growth. CPMV‐4C treated animals exhibited a ~12.2‐fold reduction in volume compared to PBS‐treated group (*p* < 0.0001). Additionally, formulated CPMV‐4P‐4C and CPMV‐40P‐4C treatments significantly inhibited tumor growth ~22.7‐fold and ~5.3 fold compared to their corresponding controls 4P (*p* < 0.0001) and 40P (*p* < 0.0001), respectively (Figure 5b). For samples incubated at 25°C, treatment with “naked” CPMV significantly inhibited tumor growth ~8‐fold compared to PBS‐treated group (*p* < 0.0001). CPMV‐4C inhibited tumor growth on average ~1.6‐fold more than CPMV‐25C, but this was not statistically significant. Formulated CPMV‐4P‐25C and CPMV‐40P‐25C treatments significantly inhibited tumor growth ~16.7‐fold and ~15.5‐fold compared to their corresponding controls 4P (*p* < 0.0001) and 40P (*p* < 0.0001), respectively (Figure 5b)—no differences in efficacy comparing the 25C “nanoarmor” formulations vs. the standard CPMV‐4C formulation were noted. For samples incubated at 37°C, CPMV‐37C only showed moderate effects on tumor growth inhibition (~2.5‐fold reduction in tumor volume compared to PBS‐treated group, *p* < 0.01). In stark contrast, formulated CPMV‐4P‐37C and CPMV‐40P‐37C treatment significantly inhibited tumor growth ~7.7‐fold and ~10.6‐fold compared to their corresponding controls 4P (*p* < 0.0001) and 40P (*p* < 0.0001), respectively (Figure 5b). Notably, with increasing incubation temperature (4°C to 25°C to 37°C), the average tumor size on day 18 among conventional CPMV‐treated mice increased from ~73 mm^3^ to ~120 mm^3^ to ~412 mm^3^, suggesting an attenuation in tumor inhibition efficacy. In the case of formulated CPMV‐4P, average tumor size increased from ~40 mm^3^ to 55 mm^3^ to 112 mm^3^, which is within error. For corresponding CPMV‐40P, average tumor size oscillated from ~159 mm^3^ to 53 mm^3^ to 83 mm^3^, which is within error (Figure 5b). All control groups exhibited rapid tumor proliferation as evidenced by tumor sizes reaching ~1000 mm^3^ by day 18. Of note, control groups did not exhibit temperature‐dependent differences in tumor growth rate or survival (Figure 5b). It is unclear why the CPMV‐40P‐4C formulation exhibited faster rates of tumor growth compared to corresponding CPMV‐40P‐25C or CPMV‐40P‐37C counterparts, as storage did not affect the structural integrity of the capsid. This isolated anomaly aside, the overall data indicate that the F127 “nanoarmor” protects CPMV at elevated temperatures and these samples maintained their biological efficacy as in situ vaccine.

The potency of CPMV and CPMV‐F127 was further highlighted by the survival data and rechallenge experiments: Survival data at day 41 indicate that at 4°C and 25°C incubation temperatures, no significant differences were observed in therapeutic efficacy among CPMV formulations (with the exception of the CPMV‐40P‐4C group, no survivors were available for the rechallenge study). At 37°C, significant differences in survival were observed: formulated CPMV‐4P‐37C and CPMV‐40P‐37C significantly improved survival compared to “naked” CPMV‐37C (*p* < 0.05). Tumor‐free survivors were rechallenged with 2.5 × 10^5^ B16F10 cells per mouse by intradermal injection on the contralateral flank (Figure 5c). At day 22, when age‐match control animals reached endpoint, all experimental groups remained tumor‐free, confirming that the CPMV‐induced antitumor immunity is durable (Figure 5d).

The survival and rechallenge data in this study suggest immune mechanisms for CPMV may largely be conserved for F127 “nanoarmor” CPMV formulations. Based on our previous studies, we have a good understanding of the CPMV mechanism of action: When CPMV is administered as an in situ vaccine, it reprograms the tumor microenvironment and primes systemic and durable antitumor immunity. Within the TME, (1) CPMV primes potent innate immune cell activation, leading to (2) secretion of inflammatory cytokines/chemokines, which leads to (3) reduction in immunosuppressive cytokines, (4) repolarization of macrophages to M1 phenotype, (5) recruitment of N1‐type neutrophils and Natural Killer (NK) cells which results in cancer cell killing and processing of tumor‐associated antigens, and finally (6) priming of adaptive antitumor responses capable of treating metastatic cancer.^2^, ^3^, ^6^ Data in this study are consistent with previous research demonstrating long‐lasting immune responses and induction of immune memory. Our prior work also highlighted that presence of RNA is critical for potency; the RNA acts as TLR7 agonist leading to type‐1 interferon signaling which potentiates the antitumor response.^7^ RNA‐free virus‐like particles of CPMV or chemically or UV‐inactivated CPMV also show potency (these formulations primarily signal through TLR2 and 4) but do not match efficacy of native CPMV.^9^, ^31^ The native CPMV is the most potent formulation identified to date and therefore the lead candidate for translational drug development.

An interesting facet of this work is that a wide range of CPMV‐F127 formulations (4–40%) was able to retain their immunogenicity. F127 is demonstrated to coat the surface of plant viruses,^12^, ^27^ however, it accomplishes this in a way which still enables PRR recognition by immune cells in vitro (Figure 4c) and in vivo (Figure 5). Notably, coating layer thickness is a function of F127 concentration (Figure 2c) but data suggest thickness‐independent PRR activation and efficacy. The utility of F127 invites applications of other pharmaceutical excipients, but a logical next step would be to screen polymer formulations in the same family of “Pluronics” with different ratios of PEO‐ to PPO‐length,^14^, ^32^ in addition to formulation pH and ionic strength. It should be noted that in addition to enabling CPMV stability against thermal degradation in storage, F127 may also confer additional benefits in vivo. In particular, the gelling phenomenon may lead to increased tumor retention of CPMV ultimately enabling to reduce the number of doses needed.

## 4 CONCLUSIONS

We have improved the aqueous thermostability of CPMV by “nanoarmoring” it with F127, thus improving its translational potential and its accessibility as a potent, cold‐chain independent cancer immunotherapy. These data strongly suggest that retention of CPMV in situ vaccination efficacy is linked to the protection of viral capsid integrity as well as the mitigation of RNA release from CPMV, which is enabled by F127 liquid or gel formulation. To the best of our knowledge, this is the first time that F127 has been used to formulate viral nanoparticles for stabilization against thermal degradation. In the process, we have generated critical information about CPMV buffer stability at elevated temperatures, which may be important for applications requiring long‐distance distribution or long‐term storage. Moreover, we have found that capsid protection does not come at the cost of immunogenicity up to 40% F127. While secondary structure data in our work provide a correlation between CPMV structural fingerprint protection and F127 formulation, the exact mechanisms of association are yet unknown. In this regard, structural biology techniques like cryo‐EM or molecular dynamics may elucidate site‐specific F127 interactions on the viral capsid surface and inform design principles to better engineer pharmaceutical excipient + viral nanoparticle formulations, which may hold merit in other disease areas as well. While outside the scope of this work, further mechanistic study at the molecular level may elucidate nuances between “nanoarmored” and traditional CPMV in situ vaccine therapy in terms of their downstream tumor biology and immunology. Notably, both formulations exhibit the ability to induce long‐lasting immune memory.

## AUTHOR CONTRIBUTIONS

**Matthew D. Shin:** Conceptualization (supporting); data curation (lead); formal analysis (lead); investigation (equal); methodology (lead); writing – original draft (lead). **Eunkyeong Jung:** Data curation (supporting); formal analysis (supporting); investigation (supporting). **Miguel Moreno‐Gonzalez:** Data curation (supporting); formal analysis (supporting); investigation (supporting). **Oscar Ortega‐Rivera:** Data curation (supporting); formal analysis (supporting); investigation (supporting). **Nicole Steinmetz:** Conceptualization (lead); funding acquisition (lead); investigation (equal); project administration (lead); supervision (lead); validation (supporting); writing – original draft (supporting); writing – review and editing (lead).

## CONFLICT OF INTEREST STATEMENT

The authors declare the following competing financial interest(s): Dr. Steinmetz is a co‐founder of, has equity in, and has a financial interest with Mosaic ImmunoEngineering Inc. Dr. Steinmetz serves as Director, Board Member, and Acting Chief Scientific Officer, and paid consultant to Mosaic. The other authors declare no potential conflict of interest.

## Supporting information


**Data S1.** Supporting Information

## Data Availability

Data are available from the authors upon reasonable request.

## References

[1] Shoeb E , Badar U , Venkataraman S , Hefferon K . Frontiers in bioengineering and biotechnology: plant nanoparticles for anti‐cancer therapy. Vaccine. 2021;9(8):830. doi:10.3390/vaccines9080830 PMC8402531 34451955

[2] Lizotte PH , Wen AM , Sheen MR , et al. In situ vaccination with cowpea mosaic virus nanoparticles suppresses metastatic cancer. Nat Nanotechnol. 2016;11(3):295‐303. doi:10.1038/nnano.2015.292 26689376 PMC4777632

[3] Mao C , Beiss V , Ho GW , Fields J , Steinmetz NF , Fiering S . In situ vaccination with cowpea mosaic virus elicits systemic antitumor immunity and potentiates immune checkpoint blockade. J Immunother Cancer. 2022;10(12):e005834. doi:10.1136/jitc-2022-005834 36460333 PMC9723958

[4] Hoopes PJ , Wagner RJ , Duval K , et al. Treatment of canine oral melanoma with nanotechnology‐based immunotherapy and radiation. Mol Pharm. 2018;15(9):3717‐3722. doi:10.1021/acs.molpharmaceut.8b00126 29613803 PMC6296751

[5] Alonso‐Miguel D , Valdivia G , Guerrera D , et al. Neoadjuvant in situ vaccination with cowpea mosaic virus as a novel therapy against canine inflammatory mammary cancer. J Immunother Cancer. 2022;10(3):e004044. doi:10.1136/jitc-2021-004044 35277459 PMC8919457

[6] Wang C , Fiering SN , Steinmetz NF . Cowpea mosaic virus promotes anti‐tumor activity and immune memory in a mouse ovarian tumor model. Adv Ther. 2019;2(5):1900003. doi:10.1002/adtp.201900003 PMC8101952 33969181

[7] Mao C , Beiss V , Fields J , Steinmetz NF , Fiering S . Cowpea mosaic virus stimulates antitumor immunity through recognition by multiple MYD88‐dependent toll‐like receptors. Biomaterials. 2021;275:120914. doi:10.1016/j.biomaterials.2021.120914 34126409 PMC8542346

[8] Wang C , Beiss V , Steinmetz NF . Cowpea mosaic virus nanoparticles and empty virus‐like particles show distinct but overlapping immunostimulatory properties. J Virol. 2019;93(21):e00129‐e00119. doi:10.1128/JVI.00129-19 31375592 PMC6803287

[9] Jung E , Mao C , Bhatia M , Koellhoffer EC , Fiering SN , Steinmetz NF . Inactivated cowpea mosaic virus for in situ vaccination: differential efficacy of formalin vs UV‐inactivated formulations. Mol Pharm. 2023;20(1):500‐507. doi:10.1021/acs.molpharmaceut.2c00744 36399598 PMC9812890

[10] Wang Q , Lin T , Tang L , Johnson JE , Finn MG . Icosahedral virus particles as addressable nanoscale building blocks. Angew Chem Int Ed. 2002;41(3):459‐462. doi:10.1002/1521-3773(20020201)41:3<459::AID-ANIE459>3.0.CO;2-O 12491378

[11] Madi M , Mioulet V , King DP , Lomonossoff GP , Montague NP . Development of a non‐infectious encapsidated positive control RNA for molecular assays to detect foot‐and‐mouth disease virus. J Virol Methods. 2015;220:27‐34. doi:10.1016/j.jviromet.2015.04.002 25864934 PMC4451496

[12] Shin MD , Hochberg JD , Pokorski JK , Steinmetz NF . Bioconjugation of active ingredients to plant viral nanoparticles is enhanced by preincubation with a Pluronic F127 polymer scaffold. ACS Appl Mater Interfaces. 2021;13(50):59618‐59632. doi:10.1021/acsami.1c13183 34890195 PMC11729460

[13] Dumortier G , Grossiord JL , Agnely F , Chaumeil JC . A review of poloxamer 407 pharmaceutical and pharmacological characteristics. Pharm Res. 2006;23(12):2709‐2728. doi:10.1007/s11095-006-9104-4 17096184

[14] Pitto‐Barry A , Barry NPE . Pluronic® block‐copolymers in medicine: from chemical and biological versatility to rationalisation and clinical advances. Polym Chem. 2014;5(10):3291‐3297. doi:10.1039/C4PY00039K

[15] Akash MSH , Rehman K , Chen S . Pluronic F127‐based thermosensitive gels for delivery of therapeutic proteins and peptides. Polym Rev. 2014;54(4):573‐597. doi:10.1080/15583724.2014.927885

[16] Stinchcomb D , Osorio JE , Wiggan O . Methods and compositions for live attenuated viruses. 2021. Accessed March 7, 2023. https://patents.google.com/patent/US11197923B2/en

[17] 샘패스쿠버 티, 필브룩마이클, 쉬들린아비바, 맥퍼슨존 엠 . Thermo‐sensitive bone growth compositions. 2015. Accessed March 7, 2023. https://patents.google.com/patent/KR20150129717A/en

[18] Abiad M , Dani B , Shalaev E . Non‐protein clostridial toxin compositions. 2021. Accessed March 7, 2023. https://patents.google.com/patent/US10973890B2/en

[19] dengue‐tetravalent‐vaccine‐live‐attenuated‐takeda‐public‐assessment‐report_en.pdf. Accessed May 28, 2023. https://www.ema.europa.eu/en/documents/outside-eu-assessment-report/dengue-tetravalent-vaccine-live-attenuated-takeda-public-assessment-report_en.pdf

[20] Wen AM , Lee KL , Yildiz I , Bruckman MA , Shukla S , Steinmetz NF . Viral nanoparticles for in vivo tumor imaging. J Vis Exp. 2012;69:4352. doi:10.3791/4352 PMC3572551 23183850

[21] Lomonossoff GP . Cowpea Mosaic Virus. Encycl Virol. 2008;569‐574. doi:10.1016/B978-012374410-4.00562-8

[22] Shinitzky M , Elitzur AC , Deamer DW . Chapter 27 – deviation from physical identity between D‐ and L‐tyrosine. In: Pályi G , Zucchi C , Caglioti L , eds. Progress in Biological Chirality. Elsevier Science Ltd; 2004:329‐337. doi:10.1016/B978-008044396-6/50029-0

[23] Chain 1, Cowpea Mosaic Virus, Small (s) Subunit – Protein – NCBI. Accessed February 27, 2023. https://www.ncbi.nlm.nih.gov/protein/29726855

[24] Yang H , Yang S , Kong J , Dong A , Yu S . Obtaining information about protein secondary structures in aqueous solution using Fourier transform IR spectroscopy. Nat Protoc. 2015;10(3):382‐396. doi:10.1038/nprot.2015.024 25654756

[25] Bishop B , Dasgupta J , Chen XS . Structure‐based engineering of papillomavirus major capsid L1: controlling particle assembly. Virol J. 2007;4:3. doi:10.1186/1743-422X-4-3 17210082 PMC1781933

[26] Baird G , Farrell C , Cheung J , Semple A , Blue J , Ahl PL . FTIR spectroscopy detects intermolecular β‐sheet formation above the high temperature Tm for two monoclonal antibodies. Protein J. 2020;39(4):318‐327. doi:10.1007/s10930-020-09907-y 32656609 PMC7387379

[27] Liu Z , Gu J , Wu M , et al. Nonionic block copolymers assemble on the surface of protein bionanoparticle. Langmuir. 2012;28(33):11957‐11961. doi:10.1021/la302588f 22877605

[28] Nejadnik MR , Olsson ALJ , Sharma PK , van der Mei HC , Norde W , Busscher HJ . Adsorption of Pluronic F‐127 on surfaces with different hydrophobicities probed by quartz crystal microbalance with dissipation. Langmuir. 2009;25(11):6245‐6249. doi:10.1021/la9001169 19374344

[29] Shukla S , Wang C , Beiss V , et al. The unique potency of cowpea mosaic virus (CPMV) in situ cancer vaccine. Biomater Sci. 2020;8(19):5489‐5503. doi:10.1039/D0BM01219J 32914796 PMC8086234

[30] Studies on the substructure of togaviruses.pdf. Accessed March 9, 2023. doi:10.1007/BF01254686.pdf

[31] Chariou PL , Beiss V , Ma Y , Steinmetz NF . In situ vaccine application of inactivated CPMV nanoparticles for cancer immunotherapy. Mater Adv. 2021;2(5):1644‐1656. doi:10.1039/D0MA00752H 34368764 PMC8323807

[32] Naharros‐Molinero A , Caballo‐González MÁ , de la Mata FJ , García‐Gallego S . Direct and reverse Pluronic micelles: design and characterization of promising drug delivery nanosystems. Pharmaceutics. 2022;14(12):2628. doi:10.3390/pharmaceutics14122628 36559122 PMC9787366

