# Supplementary material for: Pluronic F127 “nanoarmor” for stabilization of Cowpea mosaic virus immunotherapy
Source: Bioeng Transl Med. 2023 Jul 14;9(1):e10574. doi: 10.1002/btm2.10574 (PMC10771553; doi:10.1002/btm2.10574)
Supplement: Supplementary file 1 — Data S1. Supporting Information [file BTM2-9-e10574-s001.docx]

**Supporting Information**

**Pluronic F127 ‘nanoarmor’ for stabilization of Cowpea mosaic virus immunotherapy**

Matthew D. Shin^1,2^, Eunkyeong Jung^1,2^, Miguel A. Moreno-Gonzalez^1,2^, Oscar A. Ortega-Rivera^1,2^, Nicole F. Steinmetz^1,2,3,4,5,6^*

^1^Department of NanoEngineering, ^2^Center for Nano-ImmunoEngineering, ^3^Department of Bioengineering, ^4^Department of Radiology, ^5^Moores Cancer Center, ^6^Institute for Materials Discovery and Design, Department of NanoEngineering, University of California, San Diego, 9500 Gilman Dr., La Jolla CA 92093-0448, USA.

Email: [nsteinmetz@ucsd.edu](mailto:nsteinmetz@ucsd.edu)

**Supplemental Data**


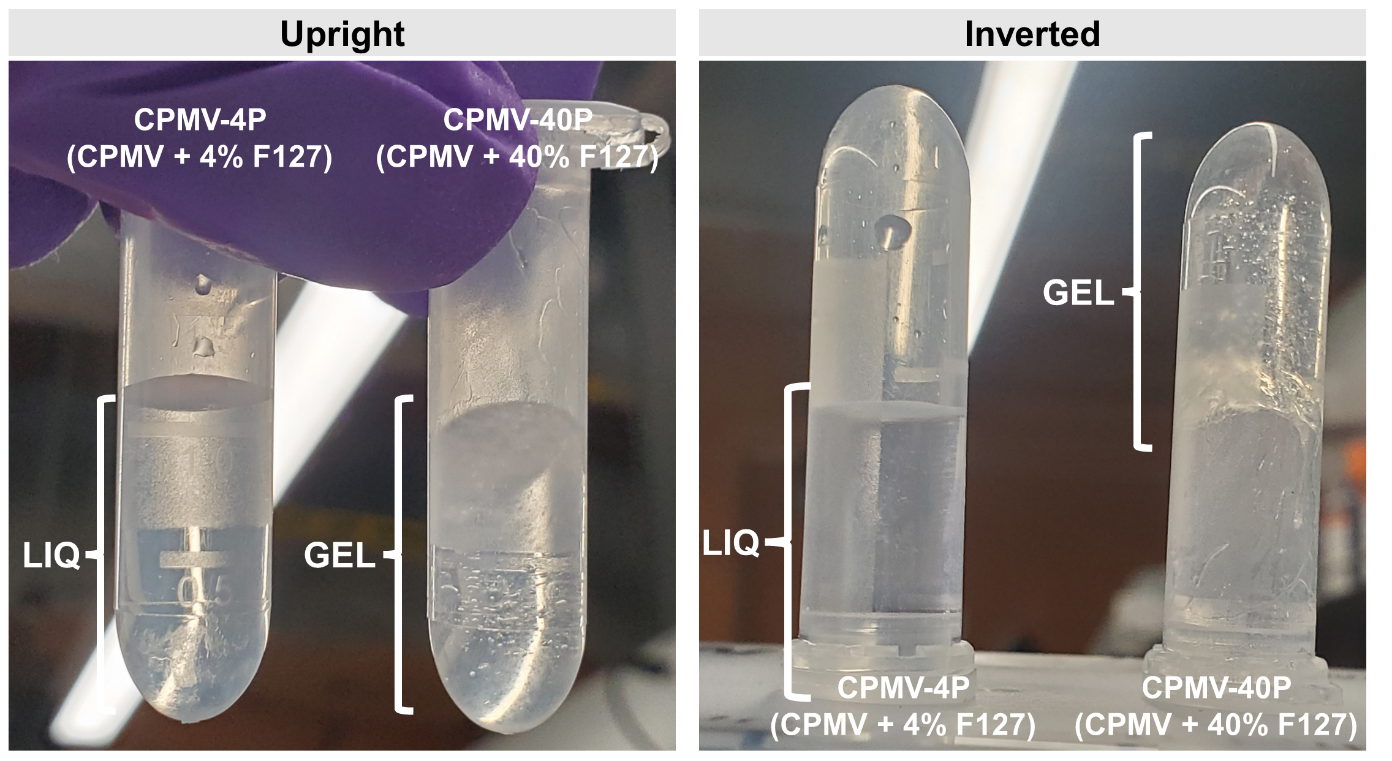


**Figure S1.** F127 “nanoarmored” CPMV formulations, CPMV-4P (on the left) and CPMV-40P (on the right), at 25 °C. Upon inversion, CPMV-4P liquid formulation displaces from its original position within the tube while the CPMV-40P gel formulation stays localized to its original position.
